# Biologics and Small Molecule Targeted Therapies for Pediatric Alopecia Areata, Psoriasis, Atopic Dermatitis, and Hidradenitis Suppurativa in the US: A Narrative Review

**DOI:** 10.3390/children11080892

**Published:** 2024-07-25

**Authors:** Robin C. Yi, Shannon K. Moran, Hannah Y. Gantz, Lindsay C. Strowd, Steven R. Feldman

**Affiliations:** 1 Center for Dermatology Research, Department of Dermatology, Wake Forest University School of Medicine, Winston-Salem, NC 27157, USA; shannon.moran@downstate.edu (S.K.M.); hgantz@wakehealth.edu (H.Y.G.); lchaney@wakehealth.edu (L.C.S.); sfeldman@wakehealth.edu (S.R.F.); 2 Department of Pathology, Wake Forest University School of Medicine, Winston-Salem, NC 27157, USA; 3 Department of Social Sciences & Health Policy, Wake Forest University School of Medicine, Winston-Salem, NC 27157, USA

**Keywords:** alopecia areata, psoriasis, atopic dermatitis, hidradenitis suppurativa, clinical trials, pediatric treatment, drug treatment, biologics, small molecules

## Abstract

Background: The management of pediatric dermatological conditions such as alopecia areata (AA), psoriasis, atopic dermatitis (AD), and hidradenitis suppurativa (HS) has significantly evolved with the introduction of biologics and small molecule targeted therapies. The advancement in understanding the immunopathogenesis of these chronic skin conditions has led to the development and approval of novel biologics and small molecule therapies. Initially approved by the United States Food and Drug Administration (FDA) for adults, most of these therapies are now being evaluated in clinical trials for safety and efficacy in adolescents and children, expanding new treatment options for pediatric patients. The role of the FDA in drug approval is multifaceted from drug inception, ensuring that research, data, and evidence show that the proposed drug is effective and safe for the intended use. Objective: The goal of this review article is to provide an overview of the recently FDA-approved and potential biologic and oral small molecule therapies in clinical trials for AA, psoriasis, AD, and HS in pediatric patients. Methods: The search for this review included keywords in ClinicalTrials.gov, PubMed, and Google Scholar for the latest research and clinical trials relevant to these conditions and treatments without the PRISMA methodology. Results: For pediatric AA, ritlecitinib is FDA-approved, while baricitinib and updacitinib are in phase 3 clinical trials for pediatric approval. The FDA-approved drugs for pediatric psoriasis include secukinumab, ustekinumab, ixekizumab, etanercept, and apremilast. Other phase 3 clinical trials for pediatric psoriasis include risankizumab, guselkumab, tildrakizumab, brodalumab, and deucravacitinib. For pediatric AD, the FDA-approved drugs are dupilumab, tralokinumab, abrocitinib, and upadacitinib, with many other drugs in phase 3 trials. Adalimumab is an FDA-approved biologic for pediatric HS, with various clinical trials ongoing for adults. The approved biologics and small molecule therapies had higher efficacy and improved safety profiles compared to traditional medications. Conclusions: With numerous ongoing trials, the success of these clinical trials could lead to their inclusion in treatment guidelines for these chronic skin conditions. Biologics and small molecule therapies offer new avenues for effective disease management, enabling personalized therapeutic interventions and improving pediatric health outcomes.

## 1. Introduction

The development of biologics and oral small molecule (OSM) therapies has revolutionized therapeutic interventions in pediatric dermatology. Chronic skin conditions such as hidradenitis suppurativa (HS), alopecia areata (AA), psoriasis, and atopic dermatitis (AD) pose challenges in management due to their chronic nature, impact on the quality of life, and prevalence in the pediatric patient population. Advancements in understanding the immunopathogenesis of these chronic skin conditions have enabled the development of novel immunomodulatory therapies, leading to a growing number of FDA-approved treatment options in recent years. The biologics and OSM therapies initially approved for adults with these chronic skin conditions are increasingly being evaluated and approved for use in pediatric patients. The use of systemic therapies requires careful consideration in pediatrics due to the developmental differences between children and adults, potential long-term effects, and the need for age-appropriate dosages and frequency. Many of these therapies are currently undergoing clinical trials to further assess their safety and efficacy in children, with the goal of expanding available treatment options. This review focuses on the currently approved and potential future biologics and OSM therapies for AA, psoriasis, AD, and HS in pediatric patients. (See Table 1).

## 2. Materials and Methods

We searched databases including ClinicalTrials.gov, PubMed, and Google Scholar for the latest research and ongoing clinical trials relevant to these conditions and treatments. Keywords included alopecia areata, psoriasis, atopic dermatitis, hidradenitis suppurativa, clinical trials, pediatric treatment, drug treatment, biologics, and small molecules. We only included phase 3 clinical trials involving biologics and oral small molecules in pediatric populations while searching for relevant clinical trials without the PRISMA methodology.

Alopecia Areata

Alopecia areata (AA) is a chronic autoimmune condition characterized by non-scarring hair loss on the scalp and other body areas. It is estimated about 2% of the general population will experience alopecia areata at some point in their lives, with approximately 50% of the patients experiencing their first episode before the age of 20 [1]. Pediatric patients diagnosed with mild AA will often be prescribed topical corticosteroids or be observed without treatment, as nearly 50% of these patients will experience spontaneous hair growth within a year [2]. The indications for biologics and OSM are for children with severe or persistent forms of AA that are refractory to first-line treatments or have a profound negative impact on quality of life [3]. Biologics and OSM are now emerging as advanced therapeutic options for managing severe or persistent forms of AA in adolescents.

FDA-Approved Small Molecule for AA

Ritlecitinib is the first approved OSM for severe AA in adolescents 12 years of age and older. This oral medication is a dual selective inhibitor of Janus kinase 3 (JAK3) and tyrosine kinase (TEC), targeting the underlying autoimmune process of AA [4]. The ALLEGRO randomized clinical trial for ritlecitinib enrolled 718 patients ages 12 and older with severe AA. The subjects who received the drug had more scalp hair coverage measured by the Severity of Alopecia Tool (SALT) compared to the placebo at 6 months [5]. The efficacy and safety of the drug were consistent amongst patient cohorts aged 12 to 17 years and 18 years of age and older [5]. The medication was well tolerated but common side effects included headaches, diarrhea, acne, urticaria, and rashes [5]. The approval of ritlecitinib provides an effective treatment option for adolescents with severe AA, where previously only adult-approved biologics and OSM therapies were available.

Phase 3 Clinical Trials, Not FDA-Approved Biologics and Small Molecule for Pediatric AA
Baricitinib

Currently approved for AA treatment in adults, baricitinib is an oral selective JAK 1/2 inhibitor [6]. This oral medication was the first small-molecule targeted therapy approved for AA in adult patients in 2022. Two randomized phase 3 clinical trials, BRAVE-AA1 and BRAVE-AA2, enrolled 1200 adults with severe AA. At 36 weeks, subjects on the drug achieved more scalp hair coverage measured by SALT compared to placebo [7,8]. The medication was generally well tolerated, though common side effects included upper respiratory infections, headache, acne, and rashes [7,8]. While baricitinib has not yet been approved for pediatric use, a new randomized phase 3 clinical trial, BRAVE-AA-PEDS, is currently active and enrolling patients aged 6 to 18 years to evaluate the safety and efficacy of baricitinib in treating severe AA in children [9].

Upadacitinib

Upadacitinib is an oral JAK1 inhibitor. A randomized phase 3 clinical trial, UP-AA, is currently ongoing to evaluate the efficacy and safety of upadacitinib in adult and adolescent subjects aged 12 to 63 years with severe AA [10]. More commonly used to treat rheumatoid and psoriatic arthritis, the outcomes of the UP-AA phase 3 trial could potentially provide a new and effective therapeutic option for both adolescents and adults affected by AA [10].

Psoriasis

Psoriasis is a chronic inflammatory skin condition that affects 1–5% of the global population. One-third of psoriasis patients experience the first symptoms of psoriasis during childhood. In patients with psoriasis aged 5 to 16 years, the health-related quality of life (QOL) is decreased by roughly one-third. Early detection and intervention prevent potential comorbidities, such as anxiety, depression, metabolic abnormalities, psoriatic arthritis (PsA), and inflammatory bowel disease. Currently, there are several FDA-approved biologics and OSM systemic treatments for pediatric psoriasis, as well as more not yet approved but currently undergoing clinical trials [11].

FDA-Approved Biologics and Small Molecule for Pediatric Psoriasis
Interleukin Inhibitors

Interleukins (ILs) are cytokines, some of which (including IL-12, IL-17, and IL-23) are involved in the inflammatory cascade. The inappropriate activation of this cascade is implicated in the pathogenesis of some diseases including psoriasis, AD, and HS. Biologic drugs that target the IL pathway can do so either through blocking the IL receptor, or by binding directly to the IL cytokine molecule [12].

Secukinumab

Secukinumab is a fully human recombinant monoclonal antibody, which selectively inhibits the IL-17A molecule, and is FDA-approved for the treatment of moderate-to-severe plaque psoriasis and PsA. It is approved for patients aged 6 years and older with moderate-to-severe plaque psoriasis who are candidates for systemic treatment or phototherapy [11].

Two phase 3 clinical trials were performed to evaluate the safety and efficacy of two dosage regimens of secukinumab compared to placebo and etanercept in pediatric patients aged 6–18 years. At week 12, secukinumab-treated patients in high and low doses achieved higher rates than placebo and etanercept in PASI-75 and PASI-90. The efficacy was maintained through week 52, demonstrating the sustained effectiveness of secukinumab in this pediatric population. In both phase 3 trials, secukinumab was well tolerated among the study population. The most common adverse events were upper respiratory infections, candida infections, and neutropenia. There were no cases of inflammatory bowel disease reported [11,13].

Ustekinumab

Ustekinumab is a monoclonal antibody that selectively inhibits IL-12 and IL-23. It is FDA-approved for the treatment of moderate-to-severe plaque psoriasis in pediatric patients aged 6 years and older who are candidates for phototherapy or systemic therapy [14].

Two randomized phase 3 clinical trials, CADMUS and CADMUS Junior, were performed to evaluate the safety and efficacy of ustekinumab in pediatric patients aged 6 to 17 years. Ustekinumab-treated patients achieved higher rates of PASI-75, PASI-90, and PASI-100 compared to those given a placebo. Ustekinumab was well tolerated, and safety events were similar to those seen in the adult population. Common adverse events included upper respiratory infections, headaches, and injection site reactions [15,16].

Ixekizumab

Ixekizumab is a monoclonal antibody that targets IL-17A and is FDA-approved for patients aged 6 years and older with moderate-to-severe plaque psoriasis [17].

A randomized phase 3 clinical trial, IXORA-PEDS, evaluated the safety and efficacy of ixekizumab in pediatric patients 6 to 18 years of age with moderate-to-severe plaque psoriasis. In IXORA-PEDS, patients achieved higher rates of PASI-75, PASI-90, and PASI-100 compared to placebo. Ixekizumab was well tolerated and the most common adverse events were nasopharyngitis, injection site reactions, and allergic reactions [17].

TNF-Alpha Inhibitors

TNF-alpha is a cytokine responsible for triggering the inflammatory cascade. Aberrant TNF-alpha activity can contribute to inflammatory conditions [18].

Etanercept

Etanercept is a TNF inhibitor, which acts as a soluble decoy receptor and binds to TNF-alpha and TNF-beta, blocking these cytokines from exerting their effects on the inflammatory cascade. It is FDA-approved for patients aged 4 years and older with moderate-to-severe plaque psoriasis [19].

Two randomized phase 3 clinical trials evaluated the safety and efficacy of etanercept in adolescents aged 4 to 17 years. Etanercept achieved PASI-75, PASI-90, and IGA 0/1 score compared to a placebo. Etanercept was well tolerated among the population studied and common adverse events seen were nasopharyngitis, upper respiratory tract infections, headaches, and injection site reactions [20].

PDE4 Inhibitors

Phosphodiesterase 4 (PDE4) can trigger the inflammatory cascade by degrading cyclic adenosine monophosphate, which in turn promotes pro-inflammatory mediator synthesis. PDE4 plays a role in some autoimmune inflammatory diseases, including psoriasis [21].

Apremilast

Apremilast is an oral selective PDE4 inhibitor and is FDA-approved for moderate-to-severe psoriasis in adults and adolescents aged 6 years and older [21].

A randomized phase 3 clinical trial, SPROUT, evaluated the efficacy and safety of apremilast for 16-weeks in patients aged 6 to 17 years with moderate-to-severe psoriasis. Subjects on the drug achieved higher rates of PASI-75 and PASI-90 compared to a placebo. Common adverse events included diarrhea, nausea, vomiting, abdominal pain, headache, and nasopharyngitis [22].

Phase 3 Clinical Trials for Pediatric Psoriasis
Interleukin Inhibitors
Risankizumab

Risankizumab is a monoclonal antibody that binds to IL-23. It is FDA-approved for adult patients with moderate-to-severe plaque psoriasis [23].

A randomized phase 3 clinical trial, OptIMMize-I, was performed to test the safety and efficacy of risankizumab compared to placebo or ustekinumab and is currently under investigation in pediatric patients aged 6 to 17 years with moderate-to-severe plaque psoriasis [24]. There is currently an active extension, OptIMMize-II, in which patients are followed for 224 weeks. The results from these trials have yet to be published [25].

Guselkumab

Guselkumab is a monoclonal antibody that binds to IL-23 and is FDA-approved for adults for the treatment of plaque psoriasis [26].

PROTOSTAR is an active, randomized phase 3 clinical trial of 120 participants and evaluated the safety and efficacy of guselkumab vs. placebo or etanercept in patients 6 to 17 years old with chronic plaque psoriasis who are candidates for phototherapy or systemic treatment. The results from this trial have yet to be published [27].

Tildrakizumab

Tildrakizumab is a monoclonal antibody that binds to the p19 subunit of IL-23, and is FDA-approved for adults with psoriasis [28]. A randomized phase 2/3 clinical trial is currently active to assess the safety and efficacy of tildrakizumab for patients with moderate-to-severe psoriasis in pediatric participants aged 12 to 17 years old, with a follow-up investigation in ages 6 to 11. The results have yet to be published [29].

Brodalumab

Brodalumab is a monoclonal antibody that inhibits IL-17A, and is FDA-approved for adults with moderate-to-severe plaque psoriasis who are candidates for systemic treatment or phototherapy [30].

A randomized phase 3 clinical trial, EMBRACE 1, is currently ongoing and evaluating the efficacy, safety, and tolerability of brodalumab compared to placebo and ustekinumab in patients 12 to 17 years old with moderate-to-severe plaque psoriasis. The results have not yet been published from this study. A follow-up phase 4 clinical trial is currently recruiting children and adolescents aged 6 to 17 years with severe plaque psoriasis to assess the safety, tolerability, and pharmacokinetics of brodalumab [31].

Tyrosine Kinase 2 Inhibitors

Tyrosine Kinase 2 (TYK2) is an intracellular kinase that regulates IL-23 and other pro-inflammatory cytokine signaling. The inappropriate activation of this cascade is implicated in psoriasis pathogenesis. Thus, TYK2 inhibitors are OSMs which bind allosterically to the regulatory domain (JH2 pseudokinase) of the TYK2 enzyme [32].

Deucravacitinib

Currently approved for adults with moderate-to-severe plaque psoriasis, deucravacitinib is an oral selective TYK2 inhibitor [32]. An ongoing randomized phase 3 clinical trial is evaluating the efficacy, safety, and pharmacokinetics of deucravacitinib in patients 4 to 17 years of age diagnosed with moderate-to-severe plaque psoriasis. For patients who completed the first aspect of the clinical trial, there is a 5-year long-term extension period to examine the longitudinal safety and tolerability of the drug. No results have been posted yet for these phase 3 clinical trials [33].

Atopic Dermatitis

Atopic dermatitis (AD) is a chronic inflammatory skin condition prevalent in childhood and adolescence that negatively impacts the quality of life. AD is a condition within the broader category of eczema, which includes other related conditions such as contact dermatitis, nummular eczema, and dyshidrotic eczema. Comprehensive treatment plans are essential for managing AD and include topical therapies and lifestyle modifications. The addition of systemic medications is indicated for moderate-to-severe disease or refractory to first-line treatment.

FDA-Approved Biologics and Small Molecules for Pediatric Atopic Dermatitis
Dupilumab

Dupilumab is a human monoclonal antibody that targets the IL-4 receptor alpha, inhibiting the IL-4 and IL-13 pathways which inhibits the pro-inflammatory cytokines and chemokines [34]. It has been approved for children aged 6 months and older with moderate-to-severe atopic dermatitis.

Two randomized phase 3 clinical trials, SOLO 1 and SOLO 2, were performed to examine the safety and efficacy of dupilimab in moderate-to-severe AD pediatric patients aged 6 months to 5 years old [35]. At week 16, patients treated with the drug achieved a higher percentage of PASI-75 compared to a placebo [35]. Adverse effects included conjunctivitis, injection-site reactions, rhinorrhea, and dental caries [35,36].

Tralokinumab

Tralokinumab is a monoclonal antibody that targets IL-13 and inhibits its ability to bind with its receptors. Currently approved for adults with AD, the FDA expanded its approval for pediatric patients aged 12 to 17 years with moderate-to-severe AD [37].

A randomized phase 3 clinical trial, ECZTRA, evaluated the safety and efficacy of tralokinumab as monotherapy in adolescents aged 12 to 17 with moderate-to-severe AD over the course of 52 weeks. Higher rates of EASI-75 were achieved in patients receiving tralokinumab 150 mg and tralokinumab 300 mg every two weeks compared to a placebo, with maintained efficacy through week 52 [37]. The medication was well tolerated and common side effects were injection-site trauma, eye and eyelid redness, and peripheral eosinophilia [37,38].

Abrocitinib

Abrocitinib is a small-molecule JAK1 inhibitor that blocks the JAK-STAT signaling pathway, which has been implicated in mediating many immune pathways in AD [39]. It is FDA-approved in adolescents aged 12 to 18. An ongoing phase 3 trial, JADE-EXTEND, is evaluating the efficacy and safety of abrocitinib in patients aged 12 or older [40]. The updated clinical results in 2023 evaluated both the 100 mg and 200 mg doses of abrocitinib with improvements in IGA, EASI-75, and PP-NRS compared to a placebo. This ongoing 92-week trial includes the primary outcome measures of safety and secondary outcome measures of efficacy using the Investigator’s Global Assessment (IGA), EASI, and Pruritus Numerical Rating Scale (NRS), among other measurements. A sub-study of JADE EXTEND will investigate potential effects on pediatric and adolescent bone health via serial knee MRI [40].

Upadacitinib

Upadacitinib is a JAK1 inhibitor and has been FDA-approved for several autoimmune and inflammatory conditions such as rheumatoid arthritis, inflammatory bowel disease (IBD), and AD in patients 12 years and older [41]. An ongoing phase 3 clinical trial is evaluating the pharmacokinetics, safety, and tolerability of oral upadacitinib in the pediatric population with severe AD. Cohorts examined in this study include pediatrics aged 6 to 12 and aged 2 to 6. The primary outcome measures include the pharmacokinetics of the drug such as plasma concentration, oral clearance, and the number of participants with treatment-emergent adverse events [42]. The results from this potential study will potentially increase the age range to 2 years of age and older.

Other Phase 3 Clinical Trials for Pediatric AD

Due to the prevalence of atopic dermatitis in the pediatric population which, when severe, can cause scarring and post-inflammatory hyperpigmentation, there are currently numerous ongoing phase 3 clinical trials for pediatrics with moderate-to-severe AD. The ongoing phase 3 clinical trials include different small molecule targets, hoping to provide alternative treatments refractory from the currently approved treatments for pediatric AD.

Two ongoing phase 3 randomized clinical trials, ROCKET-ASTRO and COAST 2, are evaluating the use of anti-OX40 antibodies, rocatinlimab and amlitelimab, in adolescents aged 12 years of age and older with moderate-to-severe AD. OX40 is a member of the TNF-alpha family and is essential in eliciting a T-cell response [43]. These studies are evaluating the primary outcome measures of the EASI score and at least a two-point reduction in baseline on the Validated Investigator’s Global Assessment for Atopic Dermatitis Score (vIGA-AD) [44,45].

Other ongoing phase 3 clinical trials are investigating the use of IL-13 inhibitors such as lebrikizumab and JAK1 inhibitors such as ivarmacitinib. Both studies are evaluating the efficacy and safety of the drugs in adolescent patients between ages 12 and 18. Both trials have the primary outcome measures of IGA score reduction of at least two points and score improvements in EASI-75 compared to placebo [46,47].

Hidradenitis Suppurativa

Hidradenitis suppurativa (HS) is a chronic inflammatory skin condition often characterized by abscesses, tracts, and nodules [48]. For children affected by HS, early and accurate diagnosis is essential for preventing progression and improving the quality of life [48]. The first-line treatment often consists of topical or oral antibiotics treatments for mild forms of HS while systemic treatments are needed for moderate-to-severe forms of HS or refractory from first-line treatments. The emerging biologics and targeted therapies have provided more treatment options for HS. The randomized clinical trials performed to test the efficacy and safety of these therapies have enrolled only adults, with the potential to extend the clinical studies to include adolescent participants. There are no recent treatment approvals for pediatric HS, as therapeutic options are still in the early phases of clinical development.

FDA-Approved Biologic for Pediatric HS
Adalimumab

Adalimumab is the first approved biologic treatment for HS in adolescents 12 years of age and older. This medication is a TNF-alpha inhibitor that reduces the underlying inflammatory disease process of HS. Two phase 3 clinical trials, PIONEER I and II, enrolled 633 adults 18 years of age and older and reduced the number of inflammatory nodules and abscesses compared to a placebo without adverse side effects [49]. Although the clinical trials for adalimumab did not enroll adolescents, the extension of its FDA approval to HS adolescent patients is largely due to its long-term safety profile in various diseases in adult and pediatric populations [50].

Phase 3 Clinical Trials for HS

There is an interest in clinical studies for the treatment of HS due to the lack of approved biologics and OSM therapies for this condition, especially in pediatrics. Numerous phase 3 clinical trials for biologics and OSM are ongoing and enrolling only adults with moderate-to-severe HS as the development and assessment of these therapies are still in the early phases to evaluate their effectiveness for HS treatment. In current phase 3 clinical trials, the mechanism of action for these emerging therapies varies amongst one another with biologics targeting specific cytokines such as TNF-alpha, IL-17A, and IL-1, and OSMs inhibiting the different pathways in JAK-STAT [51,52,53]. Many of these phase 3 clinical trials also include adult patients who have already failed TNFi therapy, making these potential therapies an alternative to the current approved treatment for HS. Depending on the success of these clinical trials, there is potential to extend these clinical studies to pediatric patients in the future.

Limitations

The focus of this review is limited to the biologics and OSM therapies available in the US, potentially missing relevant data from other regions. Additionally, the review may not encompass all of the ongoing clinical trials and emerging therapies as the literature search is based on available and published research and clinical trial data. The variability in study designs, patient populations, and endpoints across different trials presents challenges for making direct comparisons in terms of the efficacy of emerging therapies. The absence of long-term safety and efficacy data for newer approved therapies can further limit the understanding of their potential benefits and risks in pediatric age groups.

## 3. Conclusions

Before the approval of biologics and OSM therapy, the management of these chronic dermatologic conditions relied mostly on systemic immunosuppressants and anti-inflammatory agents. Most treatment options were previously limited to medications such as corticosteroids, methotrexate, cyclosporine, azathioprine, and mycophenolate. While these systemic agents offered some efficacy, they were associated with significant risks and side effects, such as immunosuppression, organ toxicity, and increased susceptibility to infections [54] (see Table 2). The variable efficacy and high safety concerns necessitate caution and routine lab monitoring, particularly for long-term use in pediatric patients [54,55,56,57,58,59,60,61,62,63,64,65,66]. Therefore, the development of biologics and OSM therapies provides alternative treatment options for these conditions, along with higher efficacy and improved safety profiles compared to previous systemic immunosuppressants. The management of these chronic skin conditions is constantly evolving, with new advancements and insights continually reshaping treatment approaches. The ongoing clinical trials offer an expansion of potential treatment options for the pediatric population with these chronic skin conditions. Within a few years, the success of these medications in clinical trials could be integrated into treatment guidelines. The emerging treatments and ongoing adaptations of guidelines can tailor personalized therapeutic interventions, improving pediatric health outcomes and quality of life.

## Figures and Tables

**Table 1:** Overview of various drugs used to treat pediatric and adult patients with chronic dermatological conditions such as alopecia areata, psoriasis, atopic dermatitis, and hidradenitis suppurativa. Details include their FDA approval status, usage in different age groups, and key phase 3 clinical trials supporting their efficacy and safety.

Chronic Condition	Drug Name	FDA Approved	Usage in Pediatrics	Usage in Adults	Key Clinical Trials
Alopecia Areata	Ritlecitinib	Yes (12 years and up)	Yes	Yes	ALLEGRO Phase 3 (>12 years)
	Baricitinib	Yes (adults only)	No	Yes	BRAVE-AA-PEDS (6–17 years)
	Upadacitinib	Yes (approved for psoriatic arthritis, AD, UC, Crohn’s, and RA)	No	No	Up-AA (>12 years)
Psoriasis	Secukinumab	Yes (6 years and older)	Yes	Yes	A2310 (6–17 years), A2311 (6–17 years)
	Ustekinumab	Yes (6 years and older)	Yes	Yes	CAMDUS (12–17 years), CAMDUS Jr (6–11 years)
	Ixekizumab	Yes (6 years and older)	Yes	Yes	IXORA-PEDS (6–17 years)
	Etanercept	Yes (4 years and older)	Yes	Yes	Phase 3 Clinical Trial [NCT00078819] (4–17 years)
	Apremilast	Yes (6 years and older)	Yes	Yes	SPROUT (6–17 years)
	Risankizumab	Yes (adults only)	No	Yes	OptIMMize-I (6–17 years, OptIMMize-II (6–17 years)
	Guselkumab	Yes (adults only)	No	Yes	PROTOSTAR (6–17 years)
	Tildrakizumab	Yes (adults only)	No	Yes	Phase 3 Clinical Trial [NCT03997786] (6–17 years)
	Brodalumab	Yes (adults only)	No	Yes	EMBRACE 1 (12–17 years)
	Deucravacitinib	Yes (adults only)	No	Yes	Phase 3 Clinical Trial [NCT0477207] (4–17 years)
Atopic Dermatitis	Dupilumab	Yes (6 months and older)	Yes	Yes	SOLO 1 (6 months–5 years), SOLO 2 (6 months–5 years)
	Tralokinumab	Yes (12 years and older)	Yes	Yes	ECZTRA (12–17 years)
	Abrocitinib	Yes (12 years and older)	Yes	Yes	JADE TEEN (12–17 years)
	Upadacitinib	Yes (12 years and older)	Yes	Yes	Measure Up 1 (>12 years), Measure Up 2 (>12 years), AD Up (>12 years)
Hidradenitis Suppurativa	Adalimumab	Yes (12 years and older)	Yes	Yes	PIONEER I (>18 years), PIONEER II (>18 years)

AA = alopecia areata; AD = atopic dermatitis; HS = hidradenitis suppurativa; UC = ulcerative colitis; RA = rheumatoid arthritis.

**Table 2:** This table compares traditional oral small molecule (OSM) and anti-metabolite systemic treatments with current biologic and OSM systemic treatments for alopecia areata, psoriasis, atopic dermatitis, and hidradenitis suppurativa in terms of efficacy and safety in pediatrics.

Chronic Condition	Drug	Efficacy in Pediatrics	Safety in Pediatrics
Alopecia Areata (AA)	Traditional OSM Treatments		
	Corticosteroids	-Complete response achieved from patients receiving intravenous or oral pulse-dosed corticosteroids (45%) versus patients receiving traditional oral corticosteroid regimens (34%) [55]	-Weight gain, cataracts, infections, hypertension, Cushingoid features, psychiatric disturbances, striae, and acne [55]
	Anti-metabolite		
	Methotrexate	-Complete response achieved in adults (44.7%) versus pediatric (11.6%) AA patients [55]	-High risk of immunosuppression, liver toxicity, and bone marrow suppression [54,55]
	JAK inhibitor		
	Ritlecitinib	-In total, 23% of patients treated with ritlecitinib 50 mg had 80% or more scalp hair coverage (SALT ≤ 20) after six months compared to placebo (1.6%) [5]	-Nausea and increased risk of upper respiratory infections [5]
Psoriasis	Anti-Metabolite		
	Methotrexate	-A total of 12/30 patients (40%) receiving methotrexate achieved PASI-75 versus 20/28 patients (71.4%) receiving biologics after 6 months. PGA was clear/almost clear (PGA 0/1) in 41 of 115 patients (35.6%) receiving methotrexate and 18 of 37 patients (48.6%) receiving biologics [58]	-Liver toxicity, bone marrow suppression, and gastrointestinal issues with long-term use [58]
	Cyclosporine	-In total, 15/38 (39.4%) psoriatic children with a median maintenance dosage per day of 3.2 mg/kg achieved PASI-75. A total of 8/38 patients (21.05%) discontinued the treatment due to laboratory anomalies or adverse events [59]	-Careful monitoring for liver and nephrotoxicity in children [59]
	Biologic and OSM Treatments		
	Secukinumab, Ustekinumab, Ixekizumab, and Etanercept	-High efficacy rates (60–95%) in achieving PASI-75, PASI-90, and PASI-100 compared to placebo (3–30%) [11,13,15,16,17,19,20]	-Upper respiratory infections, diarrhea, and potential increased risk of infections [11,13,17,20]
	Apremilast	-At 16 weeks, patients on apremilast (45.7%) achieved PASI-75 compared to placebo (16%) [22]	-Diarrhea, nausea, vomiting, abdominal pain, headache, and nasopharyngitis [22]
Atopic Dermatitis	Anti-metabolite		
	Azathioprine	-In total, 17 of 28 (61%) patients treated with azathioprine had significant improvements [60]	-Bone marrow suppression, hepatotoxicity, and increased risk of infections [60]
	Mycophenolate	-In total, 8 of 12 (66%) patients treated with mycophenolate had significant improvement [60]	-Increased gastrointestinal issues, increased risk of infections, and leukopenia [61]
	Biologic and OSM Treatments		
	Dupilumab and Tralokinumab	-At week 16, children taking dupilumab had a 75% reduction in affected body surface area [35] -At week 52, higher rates of EASI-75 were achieved in patients receiving tralokinumab 150 mg (28.6%) and tralokinumab 300 mg (27.8%) every two weeks versus placebo (6.4%) [37]	-Increased conjunctivitis, injection-site reactions, and potential increased risk of infections [35,36,37,38]
	Abrocitinib and Upadacitinib	-At week 15, patients achieved PP-NRS response with abrocitinib 200 mg (49.0%) compared with placebo (11.1%) [40] -At week 16, patients achieved EASI-75 with upadacatinib 30 mg (74%) compared to placebo (6.4%) [41,42]	-Upper respiratory infections, headache, and diarrhea [40,42]
Hidradenitis Suppurativa (HS)	Traditional OSM Treatments		
	Antibiotics	After 10 weeks, all 20 participants had some clinical improvement, with 60% achieving >50% improvement in Sartorius score [66] -A total of 108 adults with HS with Hurley stage I-III treated prospectively with tetracycline (500 mg BID), doxycycline (100 mg BID), or lymecycline (300 mg BID) achieved a mean improvement in HSS of 8.13 over a mean 4.3 months [66]	-Increased gastrointestinal issues, antibiotic resistance, and secondary infections [62]
	Corticosteroids	-In total, 14/20 (70%) HS patients achieved HiSCR [65]	-Liver toxicity, bone marrow suppression, and gastrointestinal issues [64]
	Anti-metabolite		
	Cyclosporine	-Of 18 HS patients treated with cyclosporine 2.0 to 3.5 mg/kg per day, 16 patients had slight to no improvement [66]	-Increased nephrotoxicity, hypertension, and increased infection risk [64]
	Biologic Treatment		
	Adalimumab	-Significant improvement in lesion severity and pain (41.8% and 58.9%) than those on placebo (26.0% and 27.6%) [66]	-Injection-site reactions and infections [49]

## Data Availability

No new data were created or analyzed in this study. Data sharing is not applicable to this article.
